# 10 Hz Amplitude Modulated Sounds Induce Short-Term Tinnitus Suppression

**DOI:** 10.3389/fnagi.2017.00130

**Published:** 2017-05-19

**Authors:** Patrick Neff, Jakob Michels, Martin Meyer, Martin Schecklmann, Berthold Langguth, Winfried Schlee

**Affiliations:** ^1^Neuroplasticity and Learning in the Healthy Aging Brain (HAB LAB), Department of Psychology, University of ZurichZurich, Switzerland; ^2^University Research Priority Program “Dynamics of Healthy Aging”, University of ZurichZurich, Switzerland; ^3^Department of Medicine, University of RegensburgRegensburg, Germany; ^4^Cognitive Psychology Unit, University of KlagenfurtKlagenfurt, Austria; ^5^Department of Psychiatry and Psychotherapy, University of RegensburgRegensburg, Germany

**Keywords:** tinnitus, acoustic stimulation, sound therapy, amplitude modulation, frequency modulation, residual inhibition, entrainment, alpha

## Abstract

**Objectives:** Acoustic stimulation or sound therapy is proposed as a main treatment option for chronic subjective tinnitus. To further probe the field of acoustic stimulations for tinnitus therapy, this exploratory study compared 10 Hz amplitude modulated (AM) sounds (two pure tones, noise, music, and frequency modulated (FM) sounds) and unmodulated sounds (pure tone, noise) regarding their temporary suppression of tinnitus loudness. First, it was hypothesized that modulated sounds elicit larger temporary loudness suppression (residual inhibition) than unmodulated sounds. Second, with manipulation of stimulus loudness and duration of the modulated sounds weaker or stronger effects of loudness suppression were expected, respectively.

**Methods:** We recruited 29 participants with chronic tonal tinnitus from the multidisciplinary Tinnitus Clinic of the University of Regensburg. Participants underwent audiometric, psychometric and tinnitus pitch matching assessments followed by an acoustic stimulation experiment with a tinnitus loudness growth paradigm. In a first block participants were stimulated with all of the sounds for 3 min each and rated their subjective tinnitus loudness to the pre-stimulus loudness every 30 s after stimulus offset. The same procedure was deployed in the second block with the pure tone AM stimuli matched to the tinnitus frequency, manipulated in length (6 min), and loudness (reduced by 30 dB and linear fade out). Repeated measures mixed model analyses of variance (ANOVA) were calculated to assess differences in loudness growth between the stimuli for each block separately.

**Results:** First, we found that all sounds elicit a short-term suppression of tinnitus loudness (seconds to minutes) with strongest suppression right after stimulus offset [*F*_(6, 1331)_ = 3.74, *p* < 0.01]. Second, similar to previous findings we found that AM sounds near the tinnitus frequency produce significantly stronger tinnitus loudness suppression than noise [vs. Pink noise: *t*_(27)_ = −4.22, *p* < 0.0001]. Finally, variants of the AM sound matched to the tinnitus frequency reduced in sound level resulted in less suppression while there was no significant difference observed for a longer stimulation duration. Moreover, feasibility of the overall procedure could be confirmed as scores of both tinnitus loudness and questionnaires were lower after the experiment [tinnitus loudness: *t*_(27)_ = 2.77, *p* < 0.01; Tinnitus Questionnaire: *t*_(27)_ = 2.06, *p* < 0.05; Tinnitus Handicap Inventory: *t*_(27)_ = 1.92, *p* = 0.065].

**Conclusion:** Taken together, these results imply that AM sounds, especially in or around the tinnitus frequency, may induce larger suppression than unmodulated sounds. Future studies should thus evaluate this approach in longitudinal studies and real life settings. Furthermore, the putative neural relation of these sound stimuli with a modulation rate in the EEG α band to the observed tinnitus suppression should be probed with respective neurophysiological methods.

## 1. Introduction

Subjective tinnitus is defined as “the perception of sound(s) in the absence of an external sound source” (Eggermont and Roberts, 2004; Erlandsson and Dauman, 2013) and is deemed chronic after 12 months since first occurrence (Mazurek et al., 2010). No less than 35% of the general (US) population are haunted by this phantom auditory perception at some point during their lifetime (Jastreboff, 1990). 10–15% report their tinnitus percept as being frequent or continuous and ~1–2% suffer heavily under the condition (Langguth et al., 2013). With a steadily aging demographic, tinnitus is becoming increasingly prevalent and relevant (Hoffman and Reed, 2004; Nondahl et al., 2012). Besides the tantalizing phantom sound or comorbidities like depression, stress and anxiety (Langguth et al., 2013), tinnitus also impacts daily life functions in healthy aging as impaired hearing, sound localization and speech perception can lower the quality of life in tinnitus sufferers (Moon et al., 2015; Gilles et al., 2016; Hyvärinen et al., 2016).

In the majority of cases tinnitus manifests as a single tone, ringing or noise with a definable pitch and loudness, which is perceived bilaterally or with a slight preference to one side, or alternatively lateralized to one ear (Lockwood et al., 2002). Tinnitus pitch, laterality and loudness can be therefore considered as the main (subjective) perceptual parameters of interest in addition to maskability and residual inhibition by external sounds (Henry and Meikle, 2000). Usually, tinnitus is considered to be caused by either objective (Eggermont and Roberts, 2004; Schaette and Kempter, 2006; Mazurek et al., 2010) or hidden hearing loss (Weisz et al., 2006; Schaette and McAlpine, 2011; Adjamian et al., 2012; Xiong et al., 2013), where loss of cochlear hair cells in objective hearing loss has been shown to lead to maladaptive plasticity throughout the auditory pathway and brain. Tinnitus pitch seems to average near the frequency of maximal hearing loss, especially in sufferers with pure-tone tinnitus (Schecklmann et al., 2012). Related to this maladaptive plasticity, a similarity of tinnitus to phantom limb or general phantom (pain) perception following sensory deafferentation has also been proposed (De Ridder et al., 2011). Although models of pathogenesis and physiology are still being debated and are limited by an underlying inherent heterogeneity of the disorder, it can be stated with confidence that both the inner ear and the brain are involved (Jastreboff, 1990; Eggermont and Roberts, 2004; Adjamian et al., 2009; De Ridder et al., 2011, 2014; Vanneste and De Ridder, 2012; Elgoyhen et al., 2015).

Acoustic stimulations have been used in various forms to counteract or alleviate the malicious phantom percept (Jastreboff, 2007). From a clinical routine perspective, acoustic stimulation or sound therapies are proposed as symptom-oriented treatment options besides cognitive behavioral therapy and neuromodulation or -stimulation if chronic subjective tinnitus persists after standard clinical assessment and intervention (Langguth et al., 2013). Traditionally, masking approaches using broadband or narrow-band noise, or pure tones, were established first (Feldmann, 1971; Vernon, 1977; Watanabe et al., 1997; Henry et al., 2004; Hazell and Wood, 2009). These maskers have also been administered in hearing aids (Vernon and Meikle, 2003) with slightly better effects than hearing aids without maskers as shown in a study by Henry et al. (2015). In recent times, two major acoustic stimulation techniques for long-term, daily intervention have been developed building on the model of lateral inhibition (Pantev et al., 2012; Adamchic et al., 2014). Following peripheral hearing loss, central tonotopic map reorganization and hyperactivity in regions of the reorganization responsible for the tinnitus sensation (Eggermont and Komiya, 2000; Eggermont and Roberts, 2004), lateral inhibition is theorized to counteract or reverse this maladaptive hyperactivity. Pantev and colleagues therefore proposed to apply a notch filter in a single octave band around the tinnitus frequency to music. The energy of the sound signal at the edges of the notch filter is theorized to inhibit the frequencies around the tinnitus pitch therefore reversing the maladaptive plasticity, which has been shown to be effective in long-term intervention (Okamoto et al., 2010). The width of the notch filter did not significantly influence treatment effects in a further study (Wunderlich et al., 2015b) while the spectral contrast (i.e., increased sound pressure at frequencies neighboring the filter edges) seems to improve the treatment effects as shown in a further follow up study (Stein et al., 2015). Building on similar reasoning about frequencies neighboring the tinnitus pitch and lateral inhibition, Tass and colleagues (Tass et al., 2012) established a method where sine tones are presented in a randomized fashion around the tinnitus frequency for several hours a day with similar longitudinal therapeutic effects.

While the established approaches focus on the retraining of auditory and related cortical structures in longitudinal therapeutic interventions (Pantev et al., 2012; Adamchic et al., 2014), only few studies looked at the effect of sounds on the temporary suppression of tinnitus (Roberts et al., 2006, 2008; Reavis et al., 2012) to identify possible candidates for future tinnitus sound therapies. Acoustic stimulation with amplitude modulation (AM) and frequency modulation (FM) (Reavis et al., 2012; Tyler et al., 2014) has just recently entered this line of research building on results of electrical stimulation of the cochlea (Zeng et al., 2011). The results of these studies indicate that especially AM sounds in the higher, tinnitus-relevant frequencies of 3,000–9,000 Hz produce a more pronounced tinnitus suppression during and after the stimulation compared to their unmodulated pendants or white noise. In any case, longitudinal data on efficacy and long-term as well as momentary neuroplastic alterations of continuous modulated or patterned, sounds is missing. Therefore, approaches showing efficacy and feasibility in single session experiments with short stimulation duration measuring tinnitus suppression (i.e., residual inhibition) should be tested in longitudinal, prospective placebo-controlled studies to assess long-term efficacy. While recent studies with AM and/or FM sounds, used 40 Hz for the modulation rate (Reavis et al., 2012; Tyler et al., 2014), which is known to produce the largest neural responses in auditory cortex through entrainment as shown in auditory steady-state response (ASSR) paradigms (Picton et al., 2003), no former study tested the influence of lower modulation rates in different carrier sounds, including the tinnitus pitch, for tinnitus suppression. Of special interest here, several reviewed studies in Picton et al. (2003) could also show entrainment effects for different bands including the alpha frequency band. Cortical auditory α activity has been shown to be decreased in tinnitus patients in MEG (Weisz et al., 2005; Schlee et al., 2014), EEG (Moazami-Goudarzi et al., 2010) and possibly also reduced in variability (Schlee et al., 2014). Looking at modulation depth of the stimuli and strength of (entrainment) effect as measured by EEG or MEG, several studies have reliably shown entrainment effects of monaural AM stimuli (100% modulation depth) superior to binaural AM stimuli (Picton et al., 2003; Schwarz and Taylor, 2005; Draganova et al., 2008; Becher et al., 2015). A modulation rate in the α frequency band as well as monaural stimuli with a maximized entrainment effect may therefore enable a normalization of reduced auditory α and thereby concomitantly reduce the tinnitus percept. Based on this preliminary reasoning we here investigated the effects of AM sounds in the α band for tinnitus sound therapy. Yet, the focus of this study was set on the behavioral level to proof the concept and feasibility in the absence of neurophysiological methods.

In the exploratory study at hand, we therefore tested the influence of 10 Hz AM sounds (two pure tones, noise, music and FM sounds) and unmodulated sounds (pure tone, noise) on the temporary suppression of subjective tinnitus loudness in participants with tonal tinnitus in block 1 of the experiment. We hypothesize that all sounds may elicit a short-term suppression of tinnitus loudness (seconds to minutes) with strongest suppression right after stimulus offset (Roberts et al., 2006, 2008; Reavis et al., 2012; Tyler et al., 2014). Given the different types of modulated and unmodulated sounds with frequencies in or around the actual tinnitus pitch, we expect to find differential suppression patterns between the stimuli with AM sounds possibly eliciting enhanced suppression (Reavis et al., 2012). Additionally, with the manipulation of stimulation length and loudness in block 2 of the experiment, we anticipate more pronounced or weaker effects of tinnitus loudness suppression, respectively.

## 2. Methods

### 2.1. Participants

Patients with chronic tonal tinnitus (>12 months tinnitus duration), who had consulted the multidisciplinary Tinnitus Clinic of the University of Regensburg, were included in the study if their age was between 18 and 75 years. Patients with history or presence of severe and relevant somatic, neurological, or mental disorders were excluded. Intake of psychotropic medication or ongoing participation in tinnitus therapies were further exclusion criteria. The study was approved by the Ethics Committee of the University of Regensburg (16-101-0061). All participants gave written informed consent after a comprehensive explanation of the procedures.

After signing the consent, form all participants completed the tinnitus questionnaire (TQ) (Hallam et al., 1988; Goebel and Hiller, 1994), the Tinnitus Handicap Inventory (THI) (Newman et al., 1996), and a visual analog scale (VAS) (Adamchic et al., 2012) with respect to tinnitus loudness (spanning from inaudibility to maximal imaginable loudness). The Tinnitus Sample Case History Questionnaire (TSCHQ) was used to gather clinical and demographic data of all patients (Langguth et al., 2007). Furthermore, hearing level was measured with a standard audiogram using frequencies ranging from 125 Hz to 8 kHz in octave steps with semi-octave steps between 2 and 4 (i.e., 3 kHz), and 4 and 8 kHz (i.e., 6 kHz), respectively (Madsen Midimate 622D; GN Otometrics, Denmark). Headphones used for audiometry, tinnitus matching, as well as for the stimulation procedure were quasi-linear in their frequency response over the whole audible spectrum (Sennheiser HDA 2000; Sennheiser, Germany).

Questionnaire scores and participants characteristics are listed in Table 1. The distribution of sexes in the sample was slightly skewed with 11 female and 18 male participants. 3 participants reported a purely left-sided, 2 participants a purely right-sided tinnitus. The majority of participants indicated some form of bilateral or diffuse tinnitus location, with 8 participants indicating tinnitus in both ears, 4 inside the head, 7 both ears with a tendency to the left side, and 4 with a tendency to the right side. A specific tinnitus laterality was not considered as an inclusion criterion due to the diotic presentation of the stimuli. Hearing thresholds slightly differed between ears [right side: mean = 40.63, *SD* = 13.24; left side: mean = 39.46, *SD* = 12.17; *t*_(28)_ = 2.10, *p* = 0.044].

**Table 1:** **Participants characteristics (***n*** = 29)**.

	**Mean**	**SD^a^**	**Median**	**Minimum**	**Maximum**
Age (years)	52.34	12.78	54	24	75
Tinnitus duration (months)	123.66	117.74	71	12	431
Hearing Loss (both ears, dB)	38.29	11.78	37.27	15.91	62.73
TQ^b^ total score (0–84)	39.41	14.06	40	10	69
THI^c^ total score (0–100)	43.97	18.48	44	10	92
Tinnitus loudness (%)	67.59	14.74	70	30	100
VAS^d^ loudness (0–100)	54.93	17.26	55	22	86
Tinnitus awareness (%)	66.55	26.73	60	0	100
Tinnitus frequency (matching, Hz)	5,334.77	2,904.96	6,000	911	10,500
Tinnitus loudness (matching, dBA)	45.46	14.92	43.90	23.50	81.60

a SD, Standard Deviation;

b TQ, Tinnitus Questionnaire (Goebel and Hiller, 1994);

c THI, Tinnitus Handicap Inventory (Newman et al., 1996);

d *VAS, visual analog scale*.

### 2.2. Tinnitus matching

After filling in the questionnaires and audiometry, participants were seated in front of a screen with a computer mouse and instructed for the tinnitus matching via software. The matching procedure was designed around a sine tone generator (Meyer et al., 2014) where pitch (in single Hz resolution), amplitude and laterality (panning) could be defined and controlled using MAX software (MAX 7; Cycling'74, USA). First, the loudness and lateralization of the tinnitus was roughly defined followed by the actual pitch by the study personnel (Penner and Bilger, 1992; Henry and Meikle, 2000). Participants were then made familiar with the handling of the pitch dial on the graphical user interface and informed about the possibility to adjust the tinnitus pitch in 1 Hz steps while holding down the shift key on the keyboard. Following that, participants proceeded with the actual pitch matching self-reliantly. To ensure reliability and validity of the procedure, the final pitch indicated by the participant was shifted an octave down and up and checked with the participant, respectively, to control for possible octave confusion. Finally, the matched tone was evaluated in a short discussion with the study personnel and rated on a 5 point likert scale (1 = not at all matching the tinnitus percept, 5 = perfect fit). Frequency and loudness results of the matching procedure are listed in Table 1.

### 2.3. Sound stimuli

**Figure 1:**
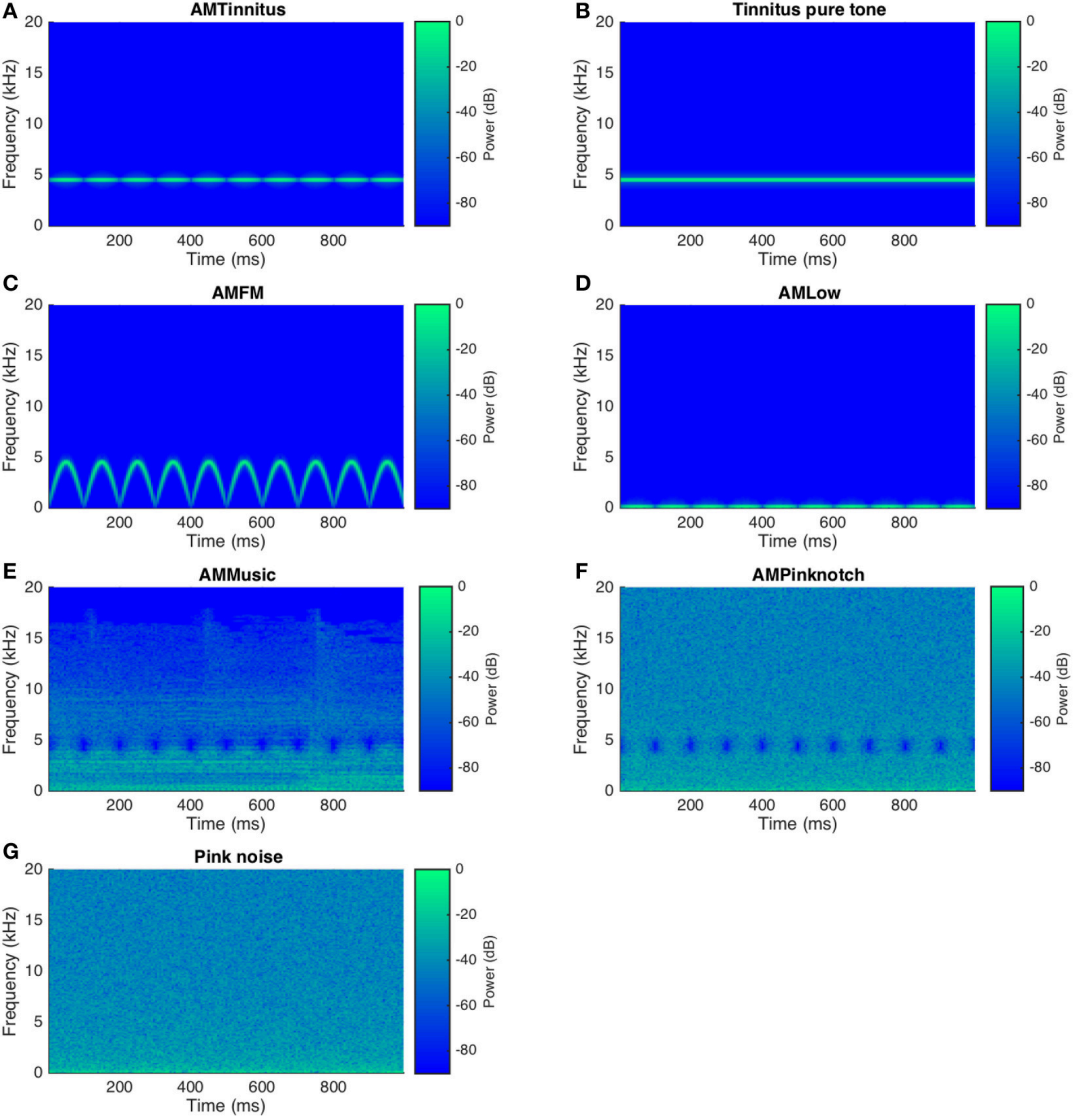
**Spectrograms of all sound stimuli (1 s snippets)**. For all of the plotted representative stimuli an arbitrary tinnitus frequency of 4,500 Hz was chosen and stimuli normalized to full digital displacement. The modulation rate was constant at 10 Hz in modulated sounds **(A,C–F)** whereas **(B,G)** represent the unmodulated stimuli. Stimulus presentation was set to 3 min for all stimuli and block 1. In block 2 AMTinnitus **(A)** underwent loudness (loudness reduction by 30 dB and linear fade out) and temporal (duration of 6 min) manipulations resulting in 4 stimuli including the standard AMTinnitus stimulus from block 1.

A set of 3 amplitude modulated, 2 notch filter amplitude modulated as well as 2 unmodulated sounds were prepared in MATLAB (Matlab R2015a; Mathworks, USA). Besides sine tones in 4 and noise in 2 conditions, a variety of popular music songs was provided to the participants out of which they could choose their favorite song for notch filter modulated presentation in one condition. A sum total of 7 acoustic stimuli or conditions with 3 min of duration was therefore produced for each participant for block 1. In the remainder of this manuscript, including tables and figures, we termed the different stimuli as follows: “AMTinnitus” for AM sounds centered at the tinnitus frequency (Figure 1A), “Tinnitus pure tone” for unmodulated sounds centered at the tinnitus frequency (Figure 1B), “AMFM” for the AM FM sound (Figure 1C), “AMLow” for AM of the 108 Hz sound (Figure 1D), “AMMusic” for the AM of musical songs (Figure 1E), “AMPinknotch” for the filter AM of pink noise (Figure 1F), and “Pink noise” for the pink noise sound (Figure 1G). For block 2, participants could choose their favorite stimulus, besides AM in the tinnitus frequency (AMTinnitus), after completing block 1. The AMTinnitus and the chosen stimulus were then manipulated in length, or loudness, or faded (linear fade out in the last minute of the stimulus) resulting in 3 conditions for two stimuli in block 2.

For AMTinnitus, a carrier sine tone was generated and amplitude modulated (100% modulation depth) with a sinusoidal function according to the following principle, where the first part of the equation represents the carrier sound and the second part the modulator. Note that the information in brackets in the legend of the equation is indicative of study-specific settings:

(1) $$\begin{array}{r} s=ca*\sin\left(2*\pi *cf*t\right)*mia*\cos\left(2*\pi *mf*t+\phi \right) \end{array}$$

where:

**Table:** 

*s*	sinusoidally amplitude modulated sound
*ca*	carrier amplitude
*cf*	carrier frequency (= tinnitus frequency)
*t*	time
*mia*	modulator index/amplitude (= 1)
*mf*	modulator frequency (= 10 Hz)
*ϕ*	phase

For the AMPinknotch and AMMusic sounds the target of the 10 Hz modulator was the notch filter amplitude. The notch filter used (Butterworth, filter order = 4) was centered around the matched tinnitus frequency with a filter bandwidth of 1 octave (Okamoto et al., 2010; Wunderlich et al., 2015a). With the filter amplitude modulation applied, the resulting sounds where rhythmically suppressed in the octave around the tinnitus frequency, giving the acoustic impression of a slight flutter in the stimulus.

For the AMFM sound, a FM sweep from 0 Hz up to the tinnitus frequency with a modulation rate of 10 Hz served as the carrier sound, which was then amplitude modulated like AMTinnitus (i.e., 100% modulation depth). AMLow with a low frequency carrier sound (108 Hz instead of tinnitus frequency) was generated analogously to AMTinnitus. Finally, the unmodulated stimuli, namely Tinnitus pure tone and Pink noise, were generated. Possible transient artifacts were avoided in the beginning and the end of the stimuli through ramping (linear fade with 100 ms window). Stimuli were then normalized in sound level and finally exported for the experimental procedure.

### 2.4. Acoustic stimulation procedure

All stimuli were presented at sound levels of 60 dB SL in block 1 (i.e., in broadband stimuli noise and music to the average hearing threshold, whereas in frequency specific stimuli the nearest frequency of the audiogram was chosen as reference for the level adjustment). For block 2, the AMTinnitus and the stimulus of choice were (1) presented for 6 min, (2) reduced in sound level (30 instead of 60 dB SL) and (3) processed with a linear sound level fade out in the last minute of the stimulus. By varying these core parameters of stimulation length and sound level in block 2, we tested differential tinnitus suppression patterns within single stimuli classes with a focus on AMTinnitus. To ensure comfort and safety of the participants, 80 dBA was the upper limit for the sound level of all stimuli. Sound level was carefully checked with an SPL meter (NTi Audio XL2; NTi Audio, Lichtenstein) before actual stimulation. Participants were reminded of the option to interrupt the procedure whenever a sound was deemed uncomfortable at any point of the experiment.

For the acoustic stimulation procedure participants were seated comfortably facing a window with a view on trees to avoid distraction and ensure calmness. No particular instruction was given to focus their attention on either the sound or tinnitus. The presentation sequence of the stimuli was randomized in the two blocks for each participant. Participants were instructed to relax during the acoustic stimulation and to rate the loudness of their tinnitus in percent, compared to the pre-stimulation loudness, after each stimulation at time points 0, 30, 60, 90, 120, 150, and 180 s. A similar approach of tinnitus loudness growth was used in the study by Reavis and colleagues (Reavis et al., 2012). However, we diverged from the former study by not measuring suppression during acoustic stimulation, having no reference tones in and after the stimulation and deploying a loudness regime tied to hearing loss with 60 dB SL (Reavis et al. (2012) presented stimuli slightly below matched tinnitus loudness). There was a short break between the blocks to maintain vigilance and comfort of the participants. At the end of the study after block 2, the VAS for tinnitus loudness and tinnitus questionnaires were again filled in by the participants. Participants were then thanked for their participation and finally dismissed.

### 2.5. Data analysis

A repeated measures mixed model analysis of variance (ANOVA) was calculated with the factors time and condition as well as a random intercept per participant to assess the effect of temporary tinnitus suppression in the loudness growth paradigm. *Post hoc* tests of the ANOVA controlled for multiple comparisons contrasting the suppression profiles between the stimuli were performed using the Tukey method. Finally, paired two-tailed *t*-tests were used to compare tinnitus questionnaire scores and tinnitus loudness VAS before and after acoustic stimulation procedure. As the 3 variables subjected to the paired comparisons were considered within an independent analysis and not part of any primary outcome statistical model or search space, we refrained from a correction for multiple comparisons (e.g., bonferroni) for this secondary analysis. R statistic toolbox with the supplementary libraries “nlme” and “lsmeans” was used for all statistical calculations (R version 3.3.2; R Foundation for Statistical Computing, Austria).

## 3. Results

### 3.1. Tinnitus loudness growth after acoustic stimulation

**Figure 2:**
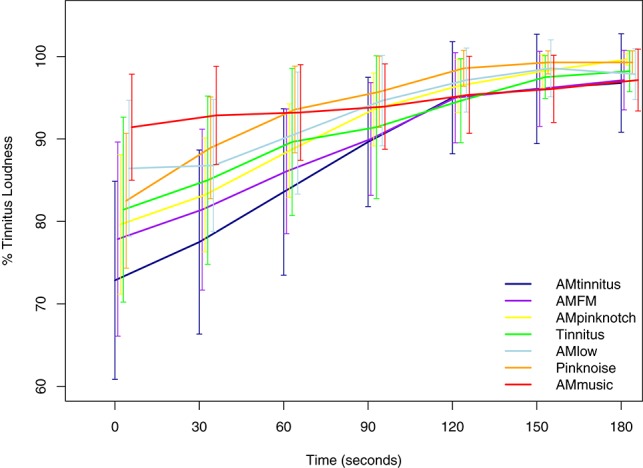
**Mean tinnitus loudness suppression after stimulus offset of all sound stimuli in block 1**. Confidence intervals at 95% are plotted for each condition and time point. Notably, after 90–120 s tinnitus loudness suppression generally diminishes and curves of the different stimuli converge. Significant differences between stimuli (conditions) are listed in Table 3.

The results of the ANOVA for the tinnitus loudness growth curves of all stimuli in block 1 are shown in Table 2 and respective corrected *post-hoc* contrasts in Table 3. Notably, there was a significant effect of condition, time, and interaction condition^*^time on the tinnitus loudness. Mean tinnitus loudness suppression curves are plotted in Figure 2.

**Table 2:** **Results of ANOVA block 1 (***n*** = 28)**.

	**numDF^a^**	**denDF^b^**	***F*-value**	***p*-value**
(Intercept)	1	1,331	2,845.28	<0.0001
Condition	6	1,331	5.40	<0.0001
Time	1	1,331	185.81	<0.0001
Condition:Time	6	1,331	3.74	0.0011

a numDF, degrees of freedom of numerator;

b *denDF, degrees of freedom of denominator*.

**Table 3:** *****Post-hoc*** contrasts block 1 (***n*** = 28, Tukey-adjusted)**.

**Contrast**	**Estimate**	***t*-value**	***p*-value**
**AMFM - AMMusic**	−**5.204**	−**3.31**	**0.016**
AMFM - AMPinknotch	−2.245	−1.43	0.786
AMFM - AMLow	−4.082	−2.60	0.127
**AMFM - Pink noise**	−**4.898**	−**3.12**	**0.031**
AMFM - AMTinnitus	1.735	1.11	0.927
AMFM - Tinnitus pure tone	−2.041	−1.30	0.852
AMMusic - AMPinknotch	2.959	1.88	0.491
AMMusic - AMLow	1.122	0.72	0.992
AMMusic - Pink noise	0.306	0.20	>0.999
**AMMusic - AMTinnitus**	**6.939**	**4.42**	**<0.0001**
AMMusic - Tinnitus pure tone	3.163	2.01	0.406
AMPinknotch - AMLow	−1.837	−1.17	0.906
AMPinknotch - Pink noise	−2.653	−1.69	0.623
AMPinknotch - AMTinnitus	3.98	2.53	0.148
AMPinknotch - Tinnitus pure tone	0.204	0.13	>0.999
AMLow - PinkNoise	−0.816	−0.52	0.999
**AMLow - AMTinnitus**	**5.816**	**3.70**	**0.004**
AMLow - Tinnitus pure tone	2.041	1.30	0.852
**Pink noise - AMTinnitus**	**6.633**	**4.22**	**0.001**
Pink noise - Tinnitus pure tone	2.857	1.82	0.535
AMTinnitus - Tinnitus pure tone	−3.776	−2.40	0.198

*Degrees of freedom = 1,331; Standard error = 1.517; Significant differences are highlighted in bold*.

*Post hoc* contrasts between each of the 7 stimuli elicited significant differences (*p* < 0.05) for AMMusic vs. AMTinnitus [*t*_(27)_ = 4.42, *p* < 0.0001], Pink noise vs. AMTinnitus [*t*_(27)_ = 4.22, *p* = 0.001], AMLow vs. AMTinnitus [*t*_(27)_ = 3.70, *p* = 0.004], AMFM vs. AMMusic [*t*_(27)_ = −3.31, *p* = 0.016], and AMFM vs. Pink Noise [*t*_(27)_ = −3.12, *p* = 0.031], respectively. These results are indicative of a pattern of enhanced tinnitus suppression of AMTinnitus and AMFM compared to Pink Noise, AMMusic, and AMLow [except AMFM vs. AMLow with *t*_(27)_ = −2.60, *p* = 0.127].

To counteract possible effects of the stimulation sequence in block 1, we furthermore tested the data for order effects with no significant results for position [*F*_(1, 1317)_ = 0.05, *p* = 0.832], condition^*^position [*F*_(6, 1317)_ = 0.94, *p* = 0.468], time^*^position [*F*_(1, 1317)_ = 3.05, *p* = 0.081], and interaction condition^*^time^*^position [*F*_(6, 1317)_ = 0.70, *p* = 0.646].

**Figure 3:**
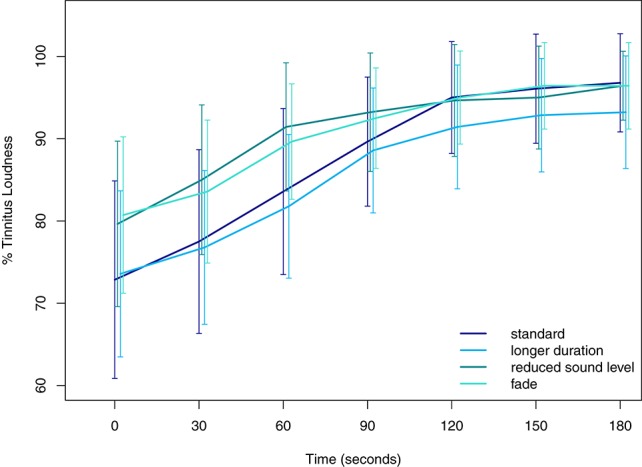
**Mean tinnitus loudness suppression after stimulus offset of AMTinnitus and its variations in block 2**. Confidence intervals at 95% are plotted for each condition and time point. Standard and longer duration of the stimulus are colored in blue whereas stimuli with reduced sound level or fade out are colored in green. Significant differences between stimuli (conditions) are listed in Table 5.

For block 2, we report the results for tinnitus loudness growth of the manipulated variations of AMTinnitus (long (6 min of duration), fade, and reduced sound level) with the addition of the data of AMTinnitus of block 1 (standard) in Table 4. *Post-hoc* contrasts are indicated in Table 5 and mean tinnitus loudness suppression curves are plotted in Figure 3. Of special interest and according to our expectations, longer stimulation (long, 6 min) resulted in a larger suppression compared to stimulations reduced in sound level [fade vs. long: *t*_(27)_ = 3.88, *p* = 0.00065; reduced sound level vs. long: *t*_(27)_ = 4.00, *p* = 0.00041] but no significant differences with the AMTinnitus stimulation for 3 min from block 1 [long vs. standard: *t*_(27)_ = −1.42, *p* = 0.486]. Furthermore, AMTinnitus elicited marginally increased suppression compared to the faded stimulus [fade vs. standard: *t*_(27)_ = 2.46, *p* = 0.067, trend] and the stimulus with reduced sound level [reduced sound level vs. standard: *t*_(27)_ = 2.57, *p* = 0.050]. The comparison of the two stimuli with manipulated sound level resulted in no significant difference [fade vs. reduced sound level: *t*_(27)_ = −0.12, *p* = 0.999].

**Table 4:** **Results of ANOVA for AMTinnitus in block 2 (***n*** = 28)**.

	**numDF^a^**	**denDF^b^**	***F*-value**	***p*-value**
(Intercept)	1	749	746.20	<0.0001
Condition	3	749	7.62	0.0001
Time	1	749	201.14	<0.0001
Condition:Time	3	749	2.70	0.0443

a numDF, degrees of freedom of numerator;

b *denDF, degrees of freedom of denominator*.

**Table 5:** *****Post-hoc*** contrasts block 2 (***n*** = 28, Tukey-adjusted)**.

**Contrast**	**Estimate**	***t*-value**	***p*-value**
Fade - Reduced sound level	−0.153	−0.12	0.999
**Fade - Long**	**5.153**	**3.88**	**0.00065**
Fade - Standard	3.265	2.46	0.067
**Reduced sound level - Long**	**5.306**	**4.00**	**0.00041**
**Reduced sound level - Standard**	**3.418**	**2.57**	**0.050**
Long - Standard	−1.887	−1.42	0.486

*Degrees of freedom = 749; Standard error = 1.328; Significant differences are highlighted in bold*.

### 3.2. Responder patterns and overall feasibility

The evaluation of the matched tinnitus pitch resulted in a mean of 4.0 (*SD* = 0.55, with 5 indicating perfect fit) highlighting the reasonable quality of the matching procedure. The response criterion for temporary tinnitus suppression was set to any suppression per stimuli (here at t0, right after the offset of the auditory stimulation) as similarly done before (Reavis et al., 2012). Applying this criterion, the following descriptive responder pattern emerges: In the AMTinnitus condition 19 out of 28 participants indicated a suppression at t0, in AMPinknotch 19/28, in AMFM 16/28, in Pink noise 16/28, in AMLow 13/28, in Tinnitus pure tone 13/28, and in AMMusic 8/20.

Differences in tinnitus loudness (VAS) and total scores of standardized questionnaires (TQ and THI) comparing assessments before and after experimental procedures are listed in Table 6 and summarized in the following: Tinnitus loudness (VAS) was significantly reduced after experimental procedures compared to the baseline assessment [*t*_(27)_ = 2.774, *p* = 0.01]. Furthermore, TQ and THI scores measuring tinnitus-related distress were also both lower after the experiment. While TQ scores are below the *p*-value threshold of *p* = 0.05, we can only report a trend for the THI [TQ: *t*_(27)_ = 2.062, *p* = 0.049; THI: *t*_(27)_ = 1.922, *p* = 0.065]. It has to be noted though, that the effects reported here are based on the possible influence of all amplitude modulated sounds as well as unmodulated “control” sounds and this secondary analysis serves safety and feasibility purposes.

**Table 6:** **Differences in tinnitus loudness and questionnaire scores before (pre) and after (post) experimental procedures**.

**Measure**	**Mean score pre**	**SD^a^ pre**	**Mean score post**	**SD post**	**df**	***t*-value**	***p*-value**
VAS loudness (mm)	54.46	17.39	48.25	17.48	27	2.774	0.01
TQ total score (0–84)	38.36	13.09	35.07	14.78	27	2.062	0.049
THI total score (0–100)	42.25	16.3	38.29	16.95	27	1.922	0.065

a *SD, Standard Deviation*.

## 4. Discussion

Acoustic stimulation or sound therapy is proposed as a main treatment option for chronic subjective tinnitus (Langguth et al., 2013). Numerous approaches for acoustic stimulation exist, be it in experimental studies (e.g., Roberts et al., 2006; Reavis et al., 2012; Hoare et al., 2014), longitudinal clinical trials (e.g., Okamoto et al., 2010; Adamchic et al., 2014), fitted hearing aids or sound players (e.g., Vernon and Meikle, 2003), mobile apps or webpages, and various user-driven self-administered forms. As of yet, there is neither an established general-purpose acoustic stimulation to abolish or reduce tinnitus nor a working strategy for subtypization of responder profiles. To further probe the field of acoustic stimulations for tinnitus therapy, the purpose of this exploratory study was to compare 10 Hz AMsounds (pure tones, noise, music and FM sounds) and unmodulated sounds (pure tone, noise) regarding their temporary suppression of tinnitus loudness in participants with tonal tinnitus.

First we found that all sounds elicit a short-term suppression of tinnitus loudness (seconds to minutes) with strongest suppression right after stimulus offset. Adding to this, feasibility of the overall procedure could be confirmed as scores of both tinnitus questionnaires as well as the VAS for tinnitus loudness were lower after the experiment. Furthermore, no adverse events or persisting increase in tinnitus loudness or distress during and after the experimental procedure were noted. Second, akin to the findings of Reavis et al. (2012), while not directly comparable (due to higher presentation loudness in our study, frequency ranges instead of matched tinnitus pitch and white noise instead of pink noise in the former study), we found that AMTinnitus and AMFM produced a significantly stronger tinnitus loudness suppression than noise.

Furthermore, both AMTinnitus and AMFM produced superior suppression than AMMusic condition with the amplitude modulated notch filter.

Finally, AMTinnitus resulted in a clearly more pronounced suppression than AMLow.

Taken together, these results imply that AM sounds, especially in or around the tinnitus frequency (i.e., AMTinnitus and AMFM, Schaette et al., 2010), may produce larger suppression than unmodulated sounds. Yet, the direct contrast between AMTinnitus and Tinnitus pure tone did not result in a significant difference, but the direction and the size of the statistical values may point to a significant contrast in future studies (see Figure 2 and Table 3). Possible cumulative effects of tinnitus suppression over the entire acoustic stimulation procedure in block 1 can be largely ruled out as there were no order effects. Third, with the manipulations of the AMTinnitus stimulus in block 2 either increasing the stimulus duration to 6 min, or reducing either overall sound level (30 dB), or fading of the stimulus in the last minute, we could partly show that these manipulations led to an altered tinnitus suppression: Standard AMTinnitus produced significantly more tinnitus suppression than both of the sound level-manipulated variations according to our expectations (i.e., reduced sound level, see Figure 3 and Table 5), yet the longer version of the very same stimulus failed to show increased overall tinnitus suppression. However, comparing the loudness growth curves of the standard AMTinnitus with the version longer in duration, there may be a difference in suppression depth from 90 s onwards after stimulation offset. While the initial suppression at 0 s seems to be in similar range in both stimuli, the longer version may sustain the suppression for a longer time as reflected in the flatter curve. This effect could be topic of possible future studies where stimulation duration undergoes respective manipulation.

Looking at the AMFM stimulus we noticed both a good suppression potential second to AMTinnitus and a promising tolerance as participants clearly preferred AMFM over all other stimuli for block 2 (10/28 chose AMFM out of the 7 alternative options). On the other hand, it is challenging to interpret these results given the lack of a direct control sound (i.e., 10 Hz FM without AM). Finally, the sounds with amplitude modulated notch filter (AMPinknotch and AMMusic) were designed to test possible short-term suppression effects of the established long-term sound therapy with notch-filtered music (Pantev et al., 2012). AMMusic clearly exhibited the least overall suppression probably due to missing energy of the sounds in and around the filtered frequency range inherent to the presented songs, as music is both spectrally and temporally highly variable in amplitude (see Figure 1E). To a lesser degree, this is also true of (pink) noise so that both of the notch-filtered AM sounds are not straight-forwardly comparable in acoustic morphology and putative suppression effects to the pure tone sounds. Furthermore, given the tonal nature of the tinnitus in participants, this result certainly was expectable. All in all, the weaker suppression effect of these filter gain modulated sounds may be due to missing energy in the critical frequency bands of the notch filter, which is not surprising given the long-term application and its putatively induced reversal of maladaptive map plasticity through residual inhibition (Pantev et al., 2012; Tass et al., 2012).

Generally, between 90 and 120 s after stimulus offset, or even earlier in some stimuli (i.e., AMMusic, Pink noise, AMPinknotch, AMLow), tinnitus loudness reaches 90% of the baseline loudness and tends to reach 100% after 180 s, which equals the stimulation duration. A similar pattern was observed by Reavis et al. (2012) in representative, individual suppression profiles, while group statistics are not performed in a comparable manner to our study. First, we did not focus on responders for statistical analyses like the previous study as all subjects, conditions and time points were included in our study. Second, no transformation on the variables or other adjustments to the raw data were performed. Yet, given the various differences in the study design of Reavis et al. (2012), namely measuring suppression during acoustic stimulation, having reference tones in and after the stimulation and applying a loudness regime slightly below matched tinnitus, results are still deemed comparable and we may substantiate the former findings that AM (and partly FM) sounds elicit better tinnitus suppression than traditional maskers (i.e., unmodulated white noise and pure tones).

### 4.1. Limitations

In the following we would like to consider some issues, which may be regarded as shortcomings of our study, while not being detrimental given the exploratory scope of this study. First, looking at the sound stimuli, unlike Reavis et al. (2012) we did not use white noise as (control) masking sounds, which may limit the interpretation of especially the contrast to AMTinnitus, as in white noise there is more sound energy in the high frequency bands where tinnitus usually manifests. Besides, there also was no direct, unmodulated control sound to AMLow and noise was not amplitude modulated over the entire audible frequency range. Future studies should therefore define respective a priori contrasts with only a single or few parameters manipulated in the stimuli to ensure optimal comparability. Second, sound presentation may be updated with consideration of tinnitus laterality (contra- vs. ipsi- vs. bilateral presentation) (Feldmann, 1971) and with related adjustments for asymmetrical hearing loss (Roberts et al., 2008), loudness weighting reconsidered (i.e., application of more detailed loudness contour curves (ISO 226) to the stimuli instead of dbA weighting), and finally matching sounds alongside the active stimuli to evaluate loudness growth independent from tinnitus (Reavis et al., 2012). Third, to both identify and analyze tinnitus subgroups as well as responder profiles, it would be advantageous to include further questionnaires to probe comorbidities and (general) quality of life (Langguth et al., 2007) and, more importantly, questionnaires elucidating personal profiles, like the NEO-PI-R (Costa and McCrae, 2008), possibly related to tolerance and acceptance of sound therapy in tinnitus. Fourth, given the behavioral nature of the current study, both neurophysiological models for cortical and subcortical responses to these stimuli and possible beneficial effects for tinnitus have to be specifically tested in fitting paradigms in future studies. Finally, in block 2, we could not test for order effects because the conditions with the stimuli chosen by the participants were deliberately left out in the analysis of the data. Given the inexistence of such order effects in block 1 and identical randomization strategies used in both blocks, we do not expect an order effect in the trimmed analysis of block 2.

### 4.2. Conclusion and outlook

Given the results of the present study in the context of previous findings, we conclude (and partly replicate) that amplitude modulated sounds with various carrier sounds in and around tinnitus frequency are feasible for short-term tinnitus suppression. With a modulation rate of 10 Hz in the EEG α band, we expect indirect neuromodulation and normalization of the endogeneous (also: individual) α rhythm which has been shown to be reduced in patients with tinnitus. Exact mechanisms of this auditory entrainment should therefore be investigated by means of respective neurophysiological methods (MEG/EEG) to test if and how auditory entrainment and possibly related tinnitus suppression is reflected by neural oscillations. Beyond that, longitudinal studies in real life should be performed to evaluate the envisioned long-term goal of this approach, namely to develop individually-customized mobile tinnitus sound therapies with aesthetically appealing sounds.

## Author contributions

PN and WS: substantial contribution to the design of the study, data analysis, drafted and revised the manuscript. JM: substantial contribution to the design of the study and data acquisition. MM: substantial contribution to the discussion of the approach and paradigm. MS and BL: drafted and revised the manuscript.

## Funding

This research was supported by by the University Research Priority Program “Dynamics of Healthy Aging” of the University of Zurich, the “Fonds zur Förderung des akademischen Nachwuchses” (FAN) des “Zürcher Universitätsvereins” (ZUNIV) and by TINNET-COST Action BM1306 “Better Understanding the Heterogeneity of Tinnitus to Improve and Develop New Treatments.”

### Conflict of interest statement

The authors declare that the research was conducted in the absence of any commercial or financial relationships that could be construed as a potential conflict of interest. The reviewer BCS and handling Editor declared their shared affiliation, and the handling Editor states that the process nevertheless met the standards of a fair and objective review.
